# Phenotypic and Genotypic Characterization of *Enterococcus* spp. Isolated from Freshwater Lakes and Rivers: Antimicrobial Resistance, Virulence Determinants and Biofilm Formation

**DOI:** 10.3390/biology15131056

**Published:** 2026-07-02

**Authors:** Katarzyna Grudlewska-Buda, Natalia Wiktorczyk-Kapischke, Anna Sędzicka, Szymon Soboń, Anna Budzyńska, Julia Czuba, Krzysztof Skowron

**Affiliations:** Department of Microbiology, Ludwik Rydygier Collegium Medicum in Bydgoszcz, Nicolaus Copernicus University in Torun, 85-094 Bydgoszcz, Poland; k.grudlewska@cm.umk.pl (K.G.-B.); anna_sedzicka@wp.pl (A.S.); szymon.sobon@o2.pl (S.S.); a.budzynska@cm.umk.pl (A.B.); julia.czuba@cm.umk.pl (J.C.); skowron238@wp.pl (K.S.)

**Keywords:** *Enterococcus* spp., freshwater, antimicrobial resistance, virulence genes, biofilm formation

## Abstract

Enterococci are environmentally persistent bacteria that are relevant to both water quality and the spread of antimicrobial resistance. This study aimed to phenotypically and genotypically characterize *Enterococcus* spp. isolated from freshwater (lakes and rivers) in north–central Poland, with a focus on antimicrobial susceptibility, virulence genes, and biofilm-forming capacity. Surface water samples were collected during the vegetation seasons of 2022 and 2023. Enterococci were isolated by culture-based methods and confirmed by MALDI-TOF MS. Antimicrobial susceptibility testing (AST) was performed using the disk diffusion method, virulence-associated genes were detected by multiplex PCR, and biofilm formation was evaluated using a crystal violet assay. In total, 96 *Enterococcus* spp. isolates representing 12 species were identified from 328 freshwater samples, with *E. faecalis* (24.0%) and *E. hirae* (21.9%) being the most frequently isolated. Thirty-one isolates (32.3%) were resistant to at least one antibiotic, and two isolates were classified as multidrug-resistant. The most prevalent virulence genes were *gelE*, *srtA*, and *hyl*. The *ace* gene was detected exclusively in *E. faecalis*. Most isolates were non-biofilm producers, while biofilm formation was confirmed in 8 strains (6 weak and 2 moderate producers), with no strong biofilm-forming strains detected. These findings suggest that freshwater ecosystems may constitute important reservoirs of antimicrobial-resistant and virulence-associated *Enterococcus* spp., underscoring the importance of continuous surveillance within the One Health framework.

## Simple Summary

*Enterococcus* spp. are present in the environment and contribute to hospital-acquired infections. In recent years, an increase in antibiotic resistance among enterococci has been observed. Surface waters may be a source of enterococci. Therefore, the aim of this study was to conduct phenotypic and genotypic characterization of *Enterococcus* spp. isolated from freshwater (lakes and rivers) in north–central Poland, with particular emphasis on antimicrobial susceptibility (disk diffusion method), virulence genes (multiplex PCR), and biofilm formation ability. Surface water samples were collected during the 2022 and 2023 growing seasons. A total of 96 *Enterococcus* spp. were isolated from 328 freshwater samples. Twelve *Enterococcus* species were confirmed, with *E. faecalis* (24.0%) and *E. hirae* (21.9%) being the most frequently isolated. Thirty-one isolates (32.3%) were resistant to at least one antibiotic, and two isolates were classified as multidrug-resistant. The most common virulence genes were *gelE*, *srtA*, and *hyl*. Most isolates did not form biofilms, whereas biofilm formation was confirmed in eight strains (six weak and two moderate producers), with no strong biofilm-forming strains detected. These findings indicate that freshwater environments can serve as reservoirs for *Enterococcus* spp. with antimicrobial resistance and virulence-associated traits, supporting the need for ongoing monitoring within the One Health framework.

## 1. Introduction

Members of the genus *Enterococcus* are Gram-positive, facultatively anaerobic cocci that naturally inhabit the gastrointestinal tracts of humans and animals but have also been widely documented in diverse environmental matrices such as soil, sediments, and freshwater systems. Their extraordinary phenotypic plasticity enables them to withstand a broad range of abiotic stressors, including temperature variation, starvation, osmotic stress, and desiccation, facilitating survival outside host environments and colonization of aquatic ecosystems [1,2].

Although enterococci are commensal in the gastrointestinal microbiota, they have emerged as significant opportunistic pathogens in clinical settings, contributing to urinary tract infections, bacteremia, infective endocarditis, and wound infections, particularly in immunocompromised individuals [3,4,5]. The pathogenic potential of *Enterococcus faecalis* and *Enterococcus faecium* is underpinned by an array of virulence determinants, such as surface adhesins (e.g., Ace, Acm), endocarditis-associated antigens (EfaAfs/EfaAfm), extracellular enzymes (e.g., gelatinase, hyaluronidase), and proteins involved in biofilm formation (e.g., Esp, Ebp, Pil) [2,6,7]. These factors facilitate adhesion to host tissues, invasion, biofilm formation, immune evasion, and persistence within both host and other environments [8].

A defining feature of enterococci is their capacity for antimicrobial resistance (AMR), including both intrinsic resistance and the acquisition of resistance determinants through horizontal gene transfer. *Enterococcus* spp. often exhibit resistance to β-lactams, aminoglycosides, macrolides, and fluoroquinolones. A particular threat is posed by vancomycin-resistant enterococci (VRE), which are increasingly causing infections in humans. The occurrence of AMR in enterococci poses a significant public health challenge, as infections caused by multidrug-resistant (MDR) strains are associated with limited therapeutic options, treatment failures, and increased healthcare burden [5,9,10,11].

Surface waters such as lakes and rivers are increasingly recognized as critical components of the global AMR landscape. Enterococci from animal and human sources are introduced to aquatic environments via wastewater discharges, agricultural runoff, stormwater, and other anthropogenic inputs, where they may persist and disseminate resistance and virulence genes [2,12,13]. For example, rivers and treated wastewater effluents have been shown to carry enterococci with similar multidrug resistance and virulence profiles, suggesting environmental waters can act as conduits for resistant strains originally associated with human activity. Enterococci also serve as practical fecal pollution indicators in water quality assessments, with ecological models demonstrating that sewage intrusion is a primary driver of elevated enterococcal counts in recreational waters (climate- and flow-based predictive modeling) [14,15,16,17].

Biofilm formation further contributes to the ecological success of enterococci in aquatic environments. Biofilms confer protection against environmental stressors and antimicrobials, and act as hotspots for horizontal gene transfer of AMR and virulence determinants, enhancing the persistence of resistant and potentially pathogenic strains [1,14,18]. *E. faecalis* has frequently been found to form more robust biofilms than *E. faecium*, and the presence of biofilm-associated genes correlates with resistance profiles and survival under environmental stress [6].

Despite the recognized importance of enterococci in environmental and clinical contexts, detailed characterization of phenotypic and genotypic traits of *Enterococcus* strains specifically isolated from natural freshwater bodies (lakes and rivers) remains comparatively limited. Many studies focus on wastewater, reclaimed water, or anthropogenically influenced river systems, but less attention has been given to open lake ecosystems and their potential role as reservoirs for resistant and virulent enterococci. These findings are in line with our previous studies on the characterization of *Enterococcus* strains isolated from wild animals, further supporting the role of natural ecosystems as reservoirs of phenotypically and genotypically diverse enterococci [19].

The aim of this study was pheno- and genotypic characterization of *Enterococcus* spp. isolated from lakes and rivers, focusing on antimicrobial susceptibility, occurrence of virulence genes, and biofilm-forming capacity. This study integrates antimicrobial susceptibility testing, molecular detection of virulence determinants, and biofilm quantification to comprehensively characterize *Enterococcus* spp. isolated from freshwater environments. Our approach aligns with the One Health framework, a concept that emerged in the early 2000s and gained international recognition through the One World, One Health initiative and the establishment of the One Health Initiative Task Force by the American Veterinary Medical Association in 2007. The framework was further refined by the One Health High-Level Expert Panel (OHHLEP), which in 2021 developed the currently accepted operational definition of One Health as an integrated approach recognizing the interdependence of human, animal, plant, and ecosystem health [20]. Accordingly, the health of a population is closely linked to the quality of its environment, including the surrounding flora and fauna. Effective assessment of microorganism transmission and factors influencing their pathogenicity, including virulence determinants and antimicrobial resistance genes, therefore requires close collaboration among specialists in biological, medical, veterinary, and genetic sciences [21,22,23]. These findings contribute to a better understanding of the dynamics of antimicrobial resistance in freshwater ecosystems, which provide suitable conditions for the spread and exchange of genetic determinants among microorganisms.

## 2. Materials and Methods

### 2.1. Study Design and Sample Collection

**Figure 1:**
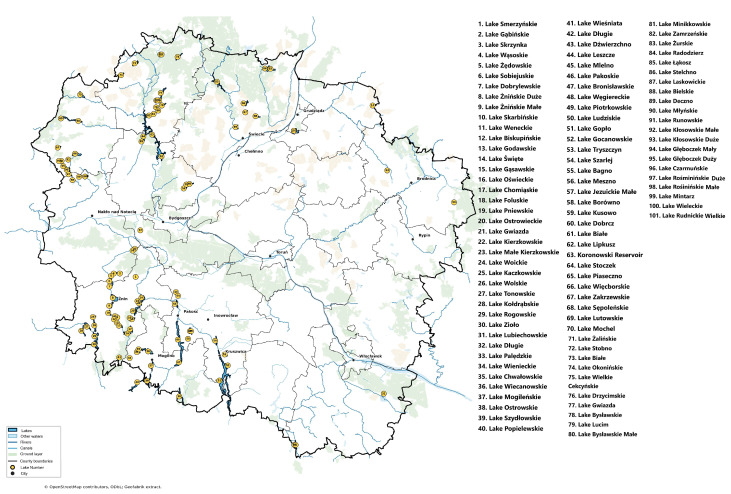
Surface water sampling sites in the Kuyavian–Pomeranian Voivodeship (lakes). *Cartographic data: OpenStreetMap, ODbL 1.0 license*.

**Figure 2:**
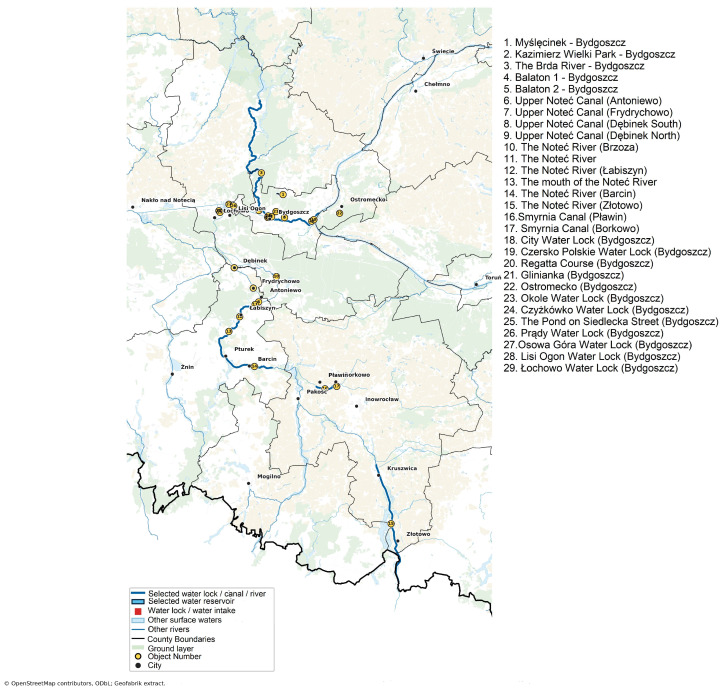
Surface water sampling sites in the Kuyavian–Pomeranian Voivodeship (other sampling sites in the Kuyavian–Pomeranian Voivodeship). *Cartographic data: OpenStreetMap, ODbL 1.0 license*.

This study was conducted on freshwater samples collected from lakes and rivers located in the Kuyavian–Pomeranian Voivodeship (north–central Poland) (Figure 1 and Figure 2). Water sampling was performed during the vegetation seasons of 2022 and 2023. Sampling sites were selected to represent different types of freshwater ecosystems and varying degrees of anthropogenic impact (Figure 1 and Figure 2). The sites included both lakes and watercourses located in different environmental settings. Samples were collected from wild bathing sites as well as organized and protected recreational areas. In addition, some water bodies were surrounded by agricultural land, whereas others were located in the vicinity of livestock farms, representing different types and intensities of anthropogenic pressure. Surface water samples were collected aseptically into sterile containers and transported to the laboratory under refrigerated conditions for immediate microbiological analysis.

### 2.2. Isolation and Identification of Enterococcus spp.

Isolation of enterococci was performed using standard culture-based methods recommended for the detection of fecal indicator bacteria. Water samples were filtered through sterile membrane filters with a pore size of 0.45 µm (Merck, Darmstadt, Germany). Following filtration, the membrane filters were placed on a selective medium, bile esculin azide agar (Merck). After incubation under aerobic conditions at 35–37 °C for 24–48 h, colonies with morphology typical for *Enterococcus* spp. were selected for further analysis.

Presumptive isolates were confirmed as members of the genus *Enterococcus* based on matrix-assisted laser desorption ionization time-of-flight mass spectrometry (MALDI-TOF MS) (Bruker, Billerica, MA, USA) according to the manufacturer’s instructions. To preserve the research material, single colonies of identified microorganisms were transferred to Eppendorf tubes with Brain Heart Infusion broth (BHI, Becton-Dickinson, Franklin Lakes, NJ, USA) and 15.0% glycerol (Avantor, Gliwice, Poland) and frozen at −80 °C.

### 2.3. Preparation of Strains

The tested strains were inoculated onto Columbia agar containing 5% sheep blood (CAB, Graso, Starogard Gdański, Poland) and incubated for 24 h at 37 °C. A single colony was subcultured onto CAB, incubated under the same conditions, and used for further testing.

### 2.4. Antimicrobial Susceptibility Testing (AST)

Antimicrobial susceptibility of the confirmed *Enterococcus* isolates was determined using the disk diffusion method on Mueller–Hinton agar (MHA) (Becton Dickinson), in accordance with the recommendations of the European Committee on Antimicrobial Susceptibility Testing (EUCAST, version 15.0). Bacterial suspensions were adjusted to the 0.5 McFarland standard and inoculated uniformly onto the agar surface.

The following antimicrobial agents were tested: ampicillin (AMP, 10 µg), imipenem (IMP, 10 µg), ciprofloxacin (CIP, 5 µg), levofloxacin (LEV, 5 µg), norfloxacin (NOR, 10 µg), gentamicin (CN, 120 µg; high-level resistance), streptomycin (S, 300 µg; high-level resistance), teicoplanin (TEC, 30 µg), vancomycin (VA, 5 µg), quinupristin–dalfopristin (QDA, 15 µg; tested only for *E. faecium*), eravacycline (ERV, 20 µg), tigecycline (TGC, 15 µg), linezolid (LZD, 10 µg), and nitrofurantoin (F, 100 µg; tested only for *E. faecalis*).

Plates were incubated at 35 °C for 18 ± 2 h; for glycopeptides, incubation was extended to 24 h. Zones of inhibition were measured in millimeters and interpreted according to EUCAST breakpoints (v. 15) [24]. *Enterococcus faecalis* ATCC 29212 was used as a quality control strain.

### 2.5. Detection of Virulence Genes by Multiplex PCR

Genotypic characterization of *Enterococcus* isolates was performed by detection of selected virulence genes using multiplex polymerase chain reaction (PCR). Genomic DNA was extracted from overnight cultures using thermal method [25].

Multiplex PCRs were designed to detect a total of ten virulence and biofilm formation-associated genes—namely *agg* (aggregation substance); *ace* (adhesion to collagen); *efaAfs* (endocarditis-associated antigen); *gelE* (gelatinase); *hyl* (hyaluronidase); *srtA* (sortase A); *ebpA*, *ebpB*, and *ebpC* (endocarditis and biofilm-associated pili); and *pil* (pili)—according to Stępień-Pyśniak et al. [26] with own modification (Table 1). The *E. faecalis* ATCC 29212 strain was used as positive control. A negative control containing nuclease-free water instead of template DNA was included in each PCR run.

### 2.6. Microtiter Plate Biofilm Production Assay

The ability of *Enterococcus* isolates to form biofilms was evaluated using a microtiter plate assay. Briefly, overnight bacterial cultures were diluted in Tryptic Soy Broth (TSB) (bioMérieux, Marcy-l’Étoile, France) and inoculated into sterile 96-well polystyrene microplates (Profilab, Warsaw, Poland). After incubation under static conditions at 37 °C for 24 h, non-adherent cells were removed by washing with phosphate-buffered saline (PBS) (Avantor). Adherent biofilms were fixed and stained with 0,1% crystal violet solution (Avantor). Then, crystal violet was removed and wells were washed with water until colorless washings. The plates were dried and then 200 μL of ethanol (Stanlab, Lublin, Poland) was added. After 5 min of shaking (400 RPM, at room temperature) absorbance at 570 nm (Abs_570_) was read in a Hidex Sense multidetection reader (HIDEX, Turku, Finland). The average Abs_570_ value, obtained from triplicates for each strain, was determined. The classification of strains as strong, moderate, or weak biofilm producers was based on a comparison of their absorbance values (A) with those of the negative control (K−), following the method described by Stepanović et al. [27]. Cut-off values were established: [K−] < [A]  ≤  2 × [K−]—weak biofilm producer; 2 × [K−] < [A]  ≤  4 × [K−]—moderate biofilm producer, 4 × [K−] < [A]—strong biofilm producer.

### 2.7. Statistical Analysis

Statistical analysis of the biofilm-related results was performed using Excel and Statistica 13.3 (TIBCO Software Inc., San Ramon, CA, USA). To assess the presence of significant differences between weak and moderate biofilm-forming strain groups, one-way analysis of variance (ANOVA) followed by Tukey’s multiple comparison test was applied. A *p*-value ≤ 0.05 was considered statistically significant.

## 3. Results

### 3.1. Occurrence of Enterococcus spp. in Freshwater Samples

During two consecutive sampling campaigns conducted in 2022 and 2023, a total of 328 freshwater samples were collected from lake and river ecosystems. Identification of the 96 *Enterococcus* isolates revealed the presence of 12 species (Table 2). In some cases, more than one strain was isolated from a single sample. The most frequently isolated species was *E. faecalis* (23; 24.0%), while the second species was *E. hirae* (21; 21.9%). One strain was isolated from each of the following species: *E. avium*, *E. columbae*, *E. moraviensis*, *E. canis* species (Table 2).

### 3.2. Antibiotic Susceptibility of Enterococcus spp. Strains

The AST results showed different resistance profiles; indeed, 31/96 (32.3%) of the isolated enterococci were resistant to at least one antimicrobial tested in the present study. Of these, 18/31 (58.1%) isolates were resistant to one antibiotic, 4/31 (12.9%) to two antibiotics, 3/96 (9.7%) to three antibiotics, and 1/31 (3.2%) *E. aquimarinus* isolate to eight antibiotics. One *E. faecalis* isolate and one *E. aquimarinus* isolate were classified as multidrug-resistant (MDR), as they were resistant to at least one antibiotic from at least three classes of antimicrobial agents active against the respective species.

The strains tested exhibited resistance to the majority of the antibiotics included in the study (Table 3). Resistance to fluoroquinolones was confirmed in one isolate each of *E. faecalis* (4.3%) and *E. aquimarinus* (50%). The remaining species were susceptible to ciprofloxacin. One (50%) *E. aquimarinus* isolate, one (4.8%) *E. hirae* isolate, and five (21.7%) *E. faecalis* isolates exhibited the high-level gentamicin resistance phenotype (HLGR). High-level streptomycin resistance (HLSR) was observed only in *E. faecalis* (10; 43.5%) and *E. hirae* (4; 19.0%). Notably, three (13.0%) isolates of *E. faecalis* described above showed the HLAR phenotype, indicating resistance to high concentrations of aminoglycosides.

For vancomycin, resistance was detected in all 19 (100.0%) *E. casseliflavus* isolates, which is associated with their intrinsic resistance to this antibiotic (VanC phenotype). Additionally, non-susceptibility was observed in two (9.5%) *E. hirae* isolates, one (50%) *E. aquimarinus* isolate, and one (4.3%) *E. faecalis* isolate. Five (31.3%) *E. faecium* isolates were resistant to quinupristin–dalfopristin. No resistance was detected to teicoplanin, linezolid, or nitrofurantoin.

### 3.3. Prevalence of Virulence-Associated Genes

**Figure 3:**
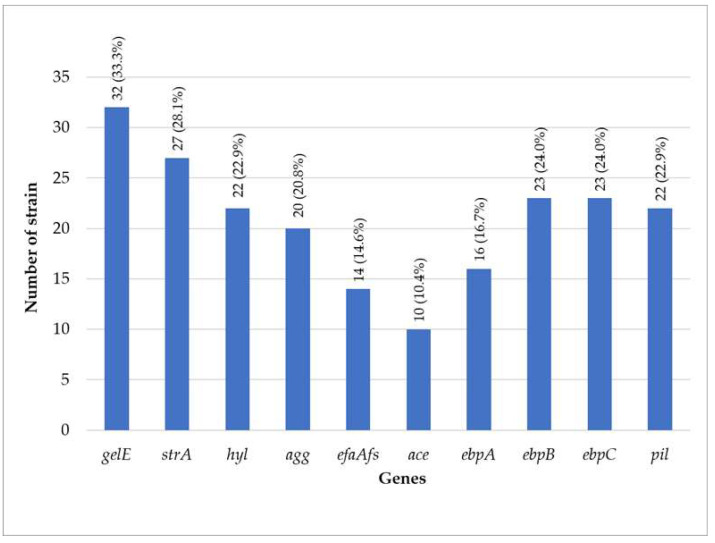
The number of *Enterococcus* spp. strains in which selected genes encoding virulence factors were confirmed.

Among the 96 isolates, the most prevalent virulence gene was *gelE*, detected in 32 (33.3%) strains, followed by *srtA* in 27 (28.1%) and *hyl* in 22 (22.9%). The genes *agg*, *efaAfs*, and *ace* were identified in 20 (20.8%), 14 (14.6%), and 10 (10.4%) isolates, respectively (Figure 3).

**Figure 4:**
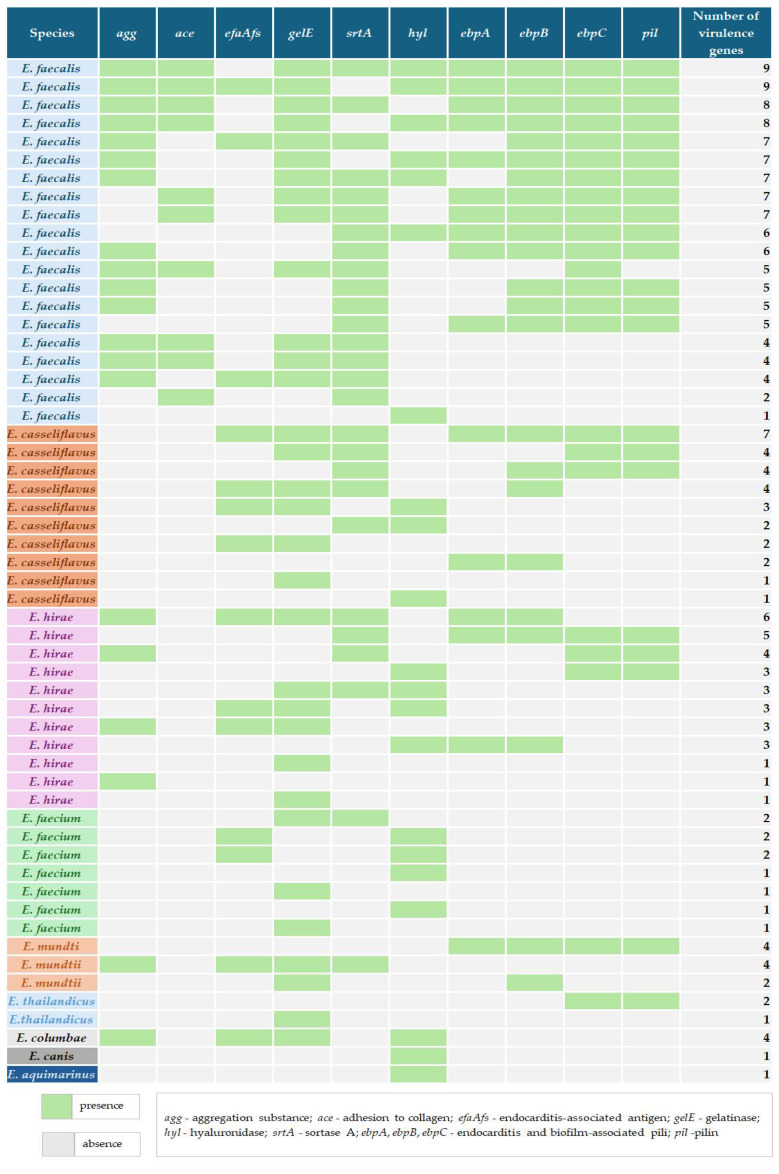
Heatmap of VG profiles from *Enterococcus* isolates investigated in the present study. Isolates are grouped according to *Enterococcus* species.

The detailed distribution of virulence genes is presented in Table 4 and Figure 4. At the species level, *E. faecalis* showed the highest prevalence of several determinants, including *gelE* (13; 56.5%), *srtA* (16; 69.6%), *agg* (14; 60.9%), and *hyl* (7; 30.4%), while *ace* was detected exclusively in this species (10; 43.5%). The *gelE* gene was also relatively frequent in *E. hirae* (6; 28.6%) and *E. casseliflavus* (6; 21.6%), whereas *hyl* was present in *E. faecium* (5; 31.3%) and *E. hirae* (4; 19.0%). The *efaAfs* gene occurred at lower frequencies across several species, including *E. casseliflavus* (4; 21%) and *E. faecalis* (3; 13%). Overall, the distribution of virulence genes varied across species, with some genes (e.g., *gelE* and *srtA*) widely distributed, while others, such as *ace*, showed a more restricted, species-specific occurrence. The least prevalent virulence determinant was *ace*, detected exclusively in *E. faecalis* isolates, where it occurred in 10/96 strains (10.4%). Analysis of the cumulative number of virulence determinants revealed that isolates carrying one virulence gene were the most common profile (13/96 isolates, 13.5%). Profiles containing seven virulence genes were identified in 6/96 isolates (6.3%). The highest virulence burden was observed exclusively in *E. faecalis*, in which isolates carrying 8–9 virulence genes were detected (4/23 isolates, 17.4%). In contrast, most non-faecalis *Enterococcus* species carried only one to four virulence determinants (Table 4 and Figure 4).

### 3.4. Biofilm-Forming Ability of Enterococcus Isolates

**Figure 5:**
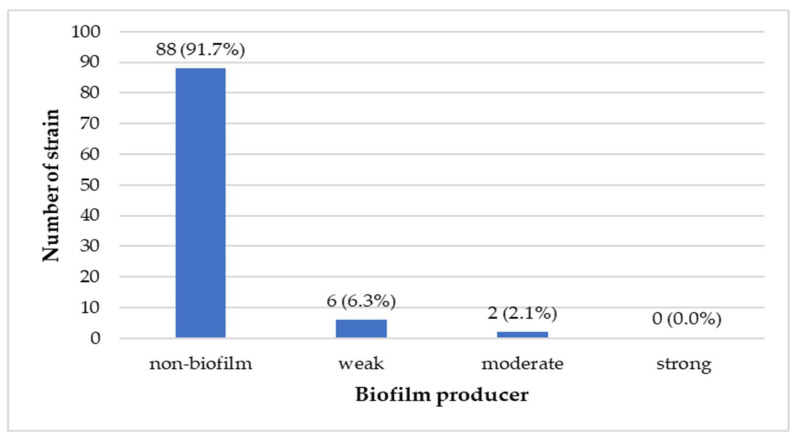
Evaluation of biofilm formation of *Enterococcus* spp. strains using crystal violet assay.

Assessment of biofilm formation using the microtiter plate assay showed that 88 isolates (91.7%) were not capable of biofilm formation (Figure 5). Biofilm-forming ability was confirmed in 8 (8.3%) strains representing the following species: *E. faecalis*, *E. casseliflavus*, *E. hirae*, *E. faecium*, and *E. columbae*. Among them, six (6.3%) strains were classified as weak biofilm producers, whereas two (2.1%) strains were identified as moderate biofilm producers. Within the studied strains, weak biofilm production was observed in two *E. faecalis* strains (8.7%), two *E. casseliflavus* strains (10.5%), one *E. faecium strain* (6.3%), and one *E. columbae* strain (100%). In contrast, moderate biofilm-forming ability was detected only in two *E. hirae* strains (9.5%) (Figure 5).

**Figure 6:**
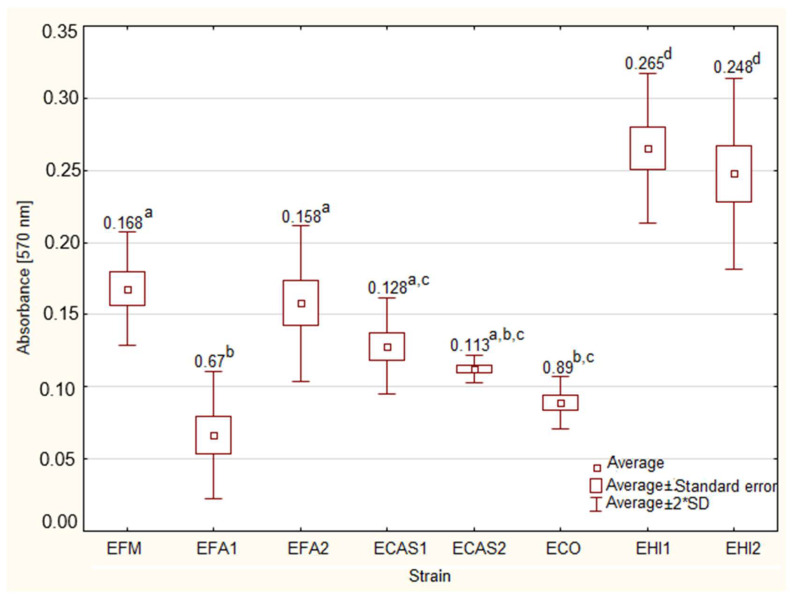
Results of the assessment of the biofilm formation ability by biofilm-forming strains (EFM—*E. faecium*, EFA—*E. faecalis*, ECAS—*E. casseliflavus*, ECO—*E. columbae*, EHI—*E. hirae*, SD—Standard deviation); a,b,c,d—values marked with different letters differ statistically significantly.

Statistically significant differences in biofilm-forming intensity were observed among the *Enterococcus* spp. strains with weak and moderate biofilm formation (*p* ≤ 0.05) (Figure 6). The mean absorbance recorded for the *E. faecium* strain was 0.168, whereas the *E. columbae* strain exhibited a mean absorbance value of 0.89. Among the *E. faecalis* strains, the mean absorbance values were 0.67 and 0.158. For *E. casseliflavus* strains, the mean absorbance values were 0.128 and 0.113. For *E. hirae* strains with moderate biofilm formation the mean absorbance value was 0.248 and 0.265.

## 4. Discussion

Bacteria belonging to the genus *Enterococcus* are commonly used as indicators of water quality and fecal contamination in aquatic environments. Maintaining high water quality, both for drinking and recreational purposes, is crucial for the protection of public health. The presence of *Enterococcus* spp. in samples collected from water reservoirs may indicate the occurrence of pathogens associated with gastrointestinal and other infections [28,29]. Various *Enterococcus* species may be isolated from surface waters depending on multiple environmental factors, including water temperature, precipitation intensity, proximity to industrial areas, and urbanization level [30]. The increasing prevalence of antibiotic-resistant bacteria constitutes a serious threat to both human and animal health, as antimicrobial therapy remains one of the primary approaches for treatment of bacterial infections. Moreover, natural environments, including surface waters, are currently considered both reservoirs and transmission routes of antimicrobial-resistant bacteria and resistance genes [31]. This observation is consistent with the One Health concept, which emphasizes the close interconnection between environmental, animal, and human health, as well as the role of environmental ecosystems in the dissemination of antimicrobial resistance.

### 4.1. Prevalence of Enterococcus spp. in Freshwater

The results obtained in the present study demonstrated considerable diversity among *Enterococcus* isolates recovered from freshwater lakes. Among the 12 identified species, *E. hirae*, *E. faecalis*, and *E. casseliflavus* were the most prevalent. Similar observations have been reported in environmental studies investigating aquatic ecosystems, where *Enterococcus* populations were shown to vary depending on anthropogenic pressure, wastewater contamination, and climatic conditions [32,33,34]. Although *E. faecalis* and *E. faecium* are traditionally regarded as the species most frequently associated with human infections, non-faecalis enterococci, including *E. hirae* and *E. casseliflavus*, are increasingly recognized as important environmental species capable of harboring virulence and antimicrobial resistance determinants [3].

### 4.2. Antibiotic Resistance Among Enterococcus spp. Strains

In our study 32.3% isolates exhibited resistance to at least one antimicrobial agent, confirming that freshwater ecosystems may serve as reservoirs of resistant enterococci. Particularly noteworthy was the identification of MDR isolates of *E. faecalis* and *E. aquimarinus*. Similar findings were described by Mbanga et al. [32], who demonstrated the presence of antimicrobial resistance genes and mobile genetic elements in environmental *Enterococcus* isolates recovered from wastewater-associated aquatic environments.

In a study conducted by Lazăr et al. [31], antimicrobial resistance profiles were evaluated among *Enterococcus* spp., including *E. faecalis*, *E. faecium*, *E. mundtii*, and *E. casseliflavus* isolates recovered from four lakes in Romania. The authors demonstrated that all 20 analyzed isolates were susceptible to penicillin, ampicillin, and levofloxacin. Similar findings were obtained in the present study. Among the 96 analyzed isolates, only one strain exhibited resistance to ampicillin, whereas resistance to levofloxacin was identified in two isolates. These observations may suggest that β-lactams and fluoroquinolones remain relatively effective therapeutic options against environmental *Enterococcus* strains.

High-level aminoglycoside resistance phenotypes, including HLGR and HLSR, were predominantly associated with *E. faecalis* isolates. This observation is clinically important because high-level resistance to aminoglycosides abolishes the synergistic effect between aminoglycosides and cell wall-active agents commonly used in enterococcal therapy. Previous studies also demonstrated that environmental enterococci may carry resistance determinants similar to those detected in clinical isolates [35,36].

Resistance to vancomycin was observed primarily among *E. casseliflavus* isolates, which is consistent with the intrinsic VanC phenotype characteristic of this species. However, resistance to vancomycin detected in several isolates of *E. hirae*, *E. faecalis*, and *E. aquimarinus* may indicate environmental dissemination of glycopeptide-resistant enterococci. Environmental transmission of vancomycin-resistant enterococci has been increasingly documented in aquatic ecosystems impacted by wastewater discharge and anthropogenic activity [37]. Importantly, none of the isolates analyzed in the present study exhibited resistance to linezolid or teicoplanin, suggesting that resistance to last-resort antimicrobials remains relatively uncommon among enterococci isolated from the investigated lakes.

In the present study, only two (2.1%) *Enterococcus* spp. isolates were classified as MDR. However, studies conducted by other authors suggest that the occurrence of MDR enterococci in aquatic environments may be substantially higher and strongly dependent on the source of isolation and geographical location. For example, Gotkowska-Płachta [14], in a study performed on *Enterococcus* spp. isolated from wastewater and the Łyna River in Olsztyn, demonstrated a very high prevalence of MDR strains. Among 283 analyzed isolates, 94.0% of wastewater-derived strains and 69.7% of river isolates were classified as MDR. Similarly, Iweriebor et al. [38], investigating *Enterococcus* spp. isolated from hospital wastewater in South Africa, reported high-level resistance to different antimicrobial agents, with several isolates exhibiting resistance to 8–10 antibiotics simultaneously. In the present study, only one isolate, identified as *E. aquimarinus*, demonstrated resistance to a comparably high number of antimicrobials, being non-susceptible to 8 out of 14 tested antibiotics. These findings indicate that the prevalence of MDR enterococci in aquatic environments may vary considerably worldwide and is likely influenced by environmental conditions, anthropogenic pressure, wastewater management practices, and the degree of exposure to antimicrobial agents. In particular, wastewater environments, especially hospital-associated effluents, appear to constitute important reservoirs of highly resistant *Enterococcus* strains [39,40].

### 4.3. Occurrence of Virulence-Associated Genes Among Enterococcus spp.

The analysis of virulence-associated genes revealed that *gelE*, *srtA*, and *hyl* were the most prevalent determinants among the investigated isolates. The predominance of *gelE* observed in the present study is in agreement with previous reports demonstrating that gelatinase is one of the most frequently detected virulence factors among environmental enterococci [35]. Gelatinase contributes to degradation of host tissues, biofilm maturation, and persistence under adverse environmental conditions, thereby increasing bacterial survival and adaptability [41]. The highest accumulation of virulence determinants was identified in *E. faecalis* isolates, which frequently carried multiple genes simultaneously, including *agg*, *gelE*, *srtA*, *ebpA*, *ebpB*, *ebpC*, and *pil*. Moreover, 8–9 virulence factors were detected in four strains of this species. These findings are consistent with previous studies demonstrating that *E. faecalis* possesses a broader repertoire of virulence-associated genes compared with other *Enterococcus* species [35,41,42]. The *ace* gene, encoding a collagen-binding protein involved in adhesion, was detected exclusively in *E. faecalis* isolates, confirming its species-associated distribution reported previously in clinical and environmental strains.

### 4.4. Biofilm Formation by Enterococcus spp. Strains

Despite the relatively frequent occurrence of virulence-associated genes related to adhesion and colonization, only a small proportion of isolates demonstrated phenotypic biofilm-forming ability. Most strains were classified as non-biofilm producers, whereas weak and moderate biofilm production was detected only in several isolates representing *E. faecalis*, *E. casseliflavus*, *E. hirae*, *E. faecium*, and *E. columbae*. None of the strains demonstrated strong biofilm-forming ability. Similar discrepancies between the presence of virulence genes and actual biofilm formation have been previously reported, suggesting that environmental conditions strongly influence gene expression and biofilm development [41].

Although the biofilm-forming ability of the investigated isolates was relatively limited, environmental biofilms remain highly important from an ecological and epidemiological perspective. Biofilm structures facilitate bacterial persistence in aquatic ecosystems and may promote horizontal transfer of antimicrobial resistance genes between environmental microorganisms [43]. Consequently, freshwater biofilms may act as reservoirs of opportunistic pathogens and antimicrobial resistance determinants, representing a potential threat to both environmental and public health.

Overall, the findings of the present study highlight the ecological significance of *Enterococcus* spp. isolated from freshwater lakes and confirm that surface waters may serve as reservoirs of potentially pathogenic and antimicrobial-resistant enterococci. The coexistence of antimicrobial resistance, virulence-associated determinants, and biofilm-related traits supports the One Health concept, emphasizing the interconnectedness of environmental, animal, and human health [37,44]. Continuous monitoring of freshwater ecosystems is therefore essential for assessing microbiological water quality and limiting the environmental dissemination of resistant bacteria.

## 5. Conclusions

The present study demonstrated that freshwater environments can harbor diverse *Enterococcus* species, including isolates exhibiting antimicrobial resistance and carrying multiple virulence-associated genes. Among the analyzed isolates, *E. faecalis* showed the highest prevalence of virulence determinants, particularly those associated with adhesion and biofilm formation. Although most isolates did not demonstrate phenotypic biofilm-forming ability, weak and moderate biofilm producers were identified, indicating that some environmental enterococci possess traits that may facilitate persistence under environmental conditions.

The coexistence of antimicrobial resistance, virulence-associated determinants, and biofilm-related traits among the analyzed isolates highlights the genetic diversity of environmental enterococci and their potential relevance as indicators of antimicrobial resistance dissemination in aquatic ecosystems. These findings contribute to a better understanding of the occurrence and distribution of resistance- and virulence-related traits in freshwater environments and support further investigation of environmental enterococci within the One Health framework. Incorporating phenotypic and genotypic characterization into environmental monitoring programs may provide additional insights into the ecology of antimicrobial resistance in surface waters.

## Figures and Tables

**Table 1:** PCR amplification conditions used for detection of virulence genes in *Enterococcus* spp.

Genes	Product Size [bp]	PCR Program
*agg*	510	Initial denaturation—94 °C/2 min 16 cycles: denaturation—94 °C/30 s annealing—58 °C/45 s extension—72 °C/45 s 19 cycles: denaturation—94 °C/30 s annealing—50 °C/45 s extension—72 °C/45 s Final extension—72 °C/7 min
*ace*	320	
*efaAfs*	705	Initial denaturation—95 °C/15 min 35 cycles: denaturation—94 °C/1 min annealing—49 °C/1 min extension—72 °C/1 min Final extension—72 °C/7 min
*gelE*	419	
*ebpC*	487	Initial denaturation—95 °C/15 min 35 cycles: denaturation—94 °C/1 min annealing—53 °C/1 min extension—72 °C/1 min Final extension—72 °C/7 min
*pil*	620	
*ebpA*	613	Initial denaturation—95 °C/15 min 35 cycles: denaturation—94 °C/1 min annealing—55 °C/1 min extension—72 °C/1 min Final extension—72 °C/7 min
*ebpB*	504	
*asa1*	375	Initial denaturation—94 °C/2 min 16 cycles: denaturation—94 °C/30 s annealing—55 °C/45 s extension—72 °C/45 s 19 cycles: denaturation—94 °C/30 s annealing—48 °C/45 s extension—72 °C/45 s Final extension—72 °C/7 min
*hyl*	276	
*srtA*	612	

**Table 2:** Distribution of *Enterococcus* species isolated from freshwater samples.

Species	2022 [n (%)]	2023 [n (%)]	Total [n (%)]
*E. faecalis*	9 (23.1)	14 (25.0)	23 (24.0)
*E. hirae*	6 (15.4)	15 (26.8)	21 (21.9)
*E. casseliflavus*	8 (20.5)	11 (19.6)	19 (19.8)
*E. faecium*	11 (28.2)	5 (8.9)	16 (16.7)
*E. mundtii*	4 (10.3)	2 (3.6)	6 (6.3)
*E. thailandicus*	0 (0.0)	3 (5.4)	3 (3.1)
*E. durans*	1 (2.6)	1 (1.8)	2 (2.1)
*E. aquimarinus*	0 (0.0)	2 (3.6)	2 (2.1)
*E. avium*	0 (0.0)	1 (1.8)	1 (1.0)
*E. columbae*	0 (0.0)	1 (1.8)	1 (1.0)
*E. moraviensis*	0 (0.0)	1 (1.8)	1 (1.0)
*E. canis*	0 (0.0)	1 (1.8)	1 (1.0)
**TOTAL**	39	57	96

**Table 3:** Antimicrobial resistance among *Enterococcus* isolates.

Antimicrobial Agent	*E. faecium* n = 16 (%)	*E. faecalis* n = 23 (%)	*E. casseliflavus* n = 19 (%)	*E. hirae* n = 21 (%)	*E. moraviensis* n = 1 (%)	*E. aquimarinus* n = 2 (%)	Total n = 96 (%) **
Ampicillin	0 (0.0)	0 (0.0)	1 (5.3)	0 (0.0)	0 (0.0)	0 (0.0)	1 (1.0)
Imipenem	0 (0.0)	0 (0.0)	1 (5.3)	0 (0.0)	0 (0.0)	0 (0.0)	1 (1.0)
Ciprofloxacin	0 (0.0)	1 (4.3)	0 (0.0)	0 (0.0)	0 (0.0)	1 (50.0)	2 (2.1)
Levofloxacin	0 (0.0)	1 (4.3)	0 (0.0)	0 (0.0)	0 (0.0)	1 (50.0)	2 (2.1)
Norfloxacin	0 (0.0)	1 (4.3)	0 (0.0)	0 (0.0)	0 (0.0)	1 (50.0)	2 (2.1)
Gentamicin	0 (0.0)	5 (21.7)	0 (0.0)	1 (4.8)	0 (0.0)	1 (50.0)	7 (7.3)
Streptomycin	0 (0.0)	10 (43.5)	0 (0.0)	4 (19.0)	0 (0.0)	0 (0.0)	14 (14.6)
Vancomycin	0 (0.0)	1 (4.3)	19 (100.0)	2 (9.5)	0 (0.0)	1 (50.0)	25 (26.0)
Eravacycline	0 (0.0)	1 (4.3)	0 (0.0)	2 (9.5)	0 (0.0)	0 (0.0)	2 (2.1)
Tigecycline	0 (0.0)	1 (4.3)	0 (0.0)	0 (0.0)	0 (0.0)	0 (0.0)	1 (1.0)
Quinupristin–dalfopristin *	5 (31.3)	NA	NA	NA	NA	NA	5 (5.2)

* Quinupristin–dalfopristin tested only for *E. faecium*. ** Only species with at least one resistant isolate are presented.

**Table 4:** Distribution of virulence-associated genes among *Enterococcus* species.

Virulence Gene	*E. faecalis* n = 23 (%)	*E. hirae* n = 21 (%)	*E. casseliflavus* n = 19 (%)	*E. faecium* n = 16 (%)	*E. mundtii * n = 6 (%)	*E. canis* n = 1 (%)	*E. columbae * n = 1 (%)	*E. thailandicus * n = 3 (%)	*E. aquimarinus* n = 2 (%)	Total n = 96 (%)
*srtA*	16 (69.6)	4 (19.0)	5 (26.3)	1 (6.25)	1 (16.7)	0 (0.0)	0 (0.0)	0 (0.0)	0 (0.0)	27 (28.1)
*efaAfs*	3 (13.0)	3 (14.3)	4 (21.0)	2 (12.5)	1 (16.7)	0 (0.0)	1 (100.0)	0 (0.0)	0 (0.0)	14 (14.6)
*ace*	10 (43.5)	0 (0.0)	0 (0.0)	0 (0.0)	0 (0.0)	0 (0.0)	0 (0.0)	0 (0.0)	0 (0.0)	10 (10.4)
*agg*	14 (60.9)	4 (19.0)	0 (0.0)	0 (0.0)	1 (16.7)	0 (0.0)	1 (100.0)	0 (0.0)	0 (0.0)	20 (20.8)
*gelE*	13 (56.5)	6 (28.6)	6 (21.6)	3 (18.8)	2 (33.3)	0 (0.0)	1 (100.0)	1 (33.3)	0 (0.0)	32 (33.3)
*hyl*	7 (30.4)	4 (19.0)	3 (15.8)	5 (31.3)	0 (0.0)	1 (100.)	1 (100.0)	0 (0.0)	1 (50.0)	22 (22.9)
*ebpA*	10 (43.5)	3 (14.3)	2 (10.5)	0 (0.0)	1 (16.7)	0 (0.0)	0 (0.0)	0 (0.0)	0 (0.0)	16 (16.7)
*ebpB*	13 (56.5)	3 (14.3)	5 (26.3)	0 (0.0)	2 (33.3)	0 (0.0)	0 (0.0)	0 (0.0)	0 (0.0)	23 (24.0)
*ebpC*	15 (65.2)	3 (14.3)	3 (15.8)	0 (0.0)	1 (16.7)	0 (0.0)	0 (0.0)	1 (33.3)	0 (0.0)	23 (24.0)
*pil*	14 (60.9)	3 (14.3)	3 (15.8)	0 (0.0)	1 (16.7)	0 (0.0)	0 (0.0)	1 (33.3)	0 (0.0)	22 (22.9)
1 VG	1 (4.3)	3 (14.3)	2 (10.5)	4 (25.0)	0 (0.0)	1 (100.0)	0 (0.0)	1 (33.3)	1 (50.0)	13 (13.5)
2 VGs	1 (4.3)	0 (0.0)	3 (15.8)	3 (18.8)	1 (16.7)	0 (0.0)	0 (0.0)	1 (33.3)	0 (0.0)	9 (9.4)
3 VGs	0 (0.0)	5 (23.8)	1 (5.3)	0 (0.0)	0 (0.0)	0 (0.0)	0 (0.0)	0 (0.0)	0 (0.0)	6 (6.2)
4 VGs	3 (13.0)	1 (4.8)	3 (15.8)	0 (0.0)	2 (33.3)	0 (0.0)	1 (100.0)	0 (0.0)	0 (0.0)	10 (10.4)
5 VGs	4 (17.4)	1 (4.8)	0 (0.0)	0 (0.0)	0 (0.0)	0 (0.0)	0 (0.0)	0 (0.0)	0 (0.0)	5 (5.2)
6 VGs	2 (8.7)	1 (4.8)	0 (0.0)	0 (0.0)	0 (0.0)	0 (0.0)	0 (0.0)	0 (0.0)	0 (0.0)	3 (3.1)
7 VGs	5 (21.7)	0 (0.0)	1 (5.3)	0 (0.0)	0 (0.0)	0 (0.0)	0 (0.0)	0 (0.0)	0 (0.0)	6 (6.2)
8–9 VGs	4 (17.4)	0 (0.0)	0 (0.0)	0 (0.0)	0 (0.0)	0 (0.0)	0 (0.0)	0 (0.0)	0 (0.0)	4 (4.2)

VG—virulence gene.

## Data Availability

The data presented in this study are available on request from the corresponding author.
